# Community health worker perspectives on a new primary health care initiative in the Eastern Cape of South Africa

**DOI:** 10.1371/journal.pone.0173863

**Published:** 2017-03-16

**Authors:** Katherine Austin-Evelyn, Miriam Rabkin, Tonderayi Macheka, Anthony Mutiti, Judith Mwansa-Kambafwile, Thomas Dlamini, Wafaa M. El-Sadr

**Affiliations:** 1 International Women’s Health Coalition, New York, New York, United States of America; 2 ICAP at Columbia University, New York, New York, United States of America; 3 Columbia University Mailman School of Public Health, New York, New York, United States of America; 4 ICAP South Africa, Johannesburg, South Africa; 5 Wits Reproductive Health and HIV Institute, Johannesburg, South Africa; 6 University of Witwatersrand, School of Clinical Medicine, Johannesburg, South Africa; 7 Eastern Cape Department of Health, Bisho, South Africa; Boston University, UNITED STATES

## Abstract

**Background:**

In 2010, South Africa’s National Department of Health launched a national primary health care (PHC) initiative to strengthen health promotion, disease prevention, and early disease detection. The strategy, called Re-engineering Primary Health Care (rPHC), aims to provide a preventive and health-promoting community-based PHC model. A key component of rPHC is the use of community-based outreach teams staffed by generalist community health workers (CHWs).

**Methods:**

We conducted focus group discussions and surveys on the knowledge and attitudes of 91 CHWs working on community-based rPHC teams in the King Sabata Dalindyebo (KSD) sub-district of Eastern Cape Province.

**Results:**

The CHWs we studied enjoyed their work and found it meaningful, as they saw themselves as agents of change. They also perceived weaknesses in the implementation of outreach team oversight, and desired field-based training and more supervision in the community.

**Conclusions:**

There is a need to provide CHWs with basic resources like equipment, supplies and transport to improve their acceptability and credibility to the communities they serve.

## Introduction

In 2010, South Africa’s National Department of Health (NDoH) launched a national primary health care (PHC) initiative to strengthen health promotion, disease prevention, and early disease detection. The strategy, called Re-engineering Primary Health Care (rPHC), aims to support a preventive and health-promoting community-based PHC model by using community-based outreach teams (known in South Africa as ward-based outreach teams or WBOTs). The WBOTs are staffed by generalist community health workers (CHWs) under the supervision of facility-based nurses, who support rPHC CHWs to provide health education, promote healthy behaviors, assess community health needs, manage minor health problems, and support linkages to health services and health facilities [1, 2].

**Fig 1:**
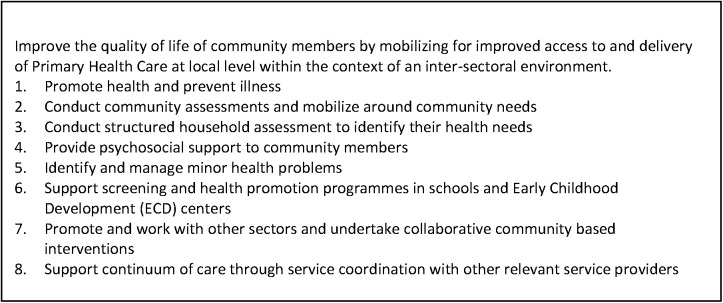
Scope of Work for CHW on rPHC Outreach Teams.

The Eastern Cape Department of Health (ECDoH) adopted the national rPHC strategy in 2011, concentrating initial efforts on launching PHC outreach teams. Starting in 2012, ECDoH piloted rPHC activities in three of its 26 sub-districts. The initial plan was for each health clinic in the pilot areas to have at least one outreach team comprised of a professional nurse and six CHWs, with each team responsible for 1,500 surrounding households with a population of approximately 6,000 people. In 2012, the DOH developed a formal scope of work (SOW) for rPHC CHW; key elements are summarized in Fig 1.

The Eastern Cape Province has had a long history of CHW programs, but prior to the launch of rPHC, these were largely run by non-governmental organizations (NGOs) and were focused on specific diseases, such as HIV, tuberculosis or maternal health services [3–5]. Over time, this resulted in diverse CHW programs with varying eligibility criteria, job descriptions and training. Novel elements of the rPHC teams included integrating generalist CHWs into the public health care system, standardizing CHW roles and training, and establishing team leadership by facility-based professional nurses.

The rural OR Tambo district is amongst the most disadvantaged districts in South Africa and ranked 49^th^ out of 50 in a 2014 assessment of district health system performance [6]. The district is also a pilot site for South Africa’s National Health Insurance program. Within OR Tambo, the King Sabato Dalindyebo (KSD) sub-district has a population of 451,710 people served by four hospitals, five health centers, and 40 clinics [7].More than half of KSD residents (55%) live below South Africa’s poverty level, 35% are dependent on social grants, and the official unemployment rate is 38.3%. The KSD human development index (HDI) is 0.49, considerably lower than the national HDI of 0.66, indicating that the sub-district falls well below the national average in this composite indicator of life expectancy, education and per capita income [7].

In KSD, few de novo staff were hired to support the rPHC initiative; most were shifted from existing programs and projects within the sub district. Due to a shortage of CHWs, the scarcity of nurses at KSD health facilities, and the competing demands on nurses’ time, plans for the configuration of rPHC outreach teams changed during the early stages of implementation; the number and size of teams was reduced and in some cases a single facility-based nurse supervised multiple outreach teams.

CHW training was coordinated by the Eastern Cape Regional Training Center, using a curriculum developed by the Foundation for Professional Development and endorsed by the ECDoH. Regional Training Center records include pre-test and post-test data for 330 CHWs trained in rPHC in 2012–2013. Average pre-test and post-test scores on the written examination were 76% and 79% respectively; the median pre-test score was 76%, rising to 80% on the post-test.

## Materials and methods

### Study design

In 2014, we conducted a formative process evaluation and strategic assessment of rPHC implementation in KSD sub-district covering the time period from 2012–2013 [8]. For the current analysis, we examine data from CHW focus group discussions and surveys of their knowledge, attitudes and practices. Using a conceptual framework proposed by Pallas et al. [9] to explore CHW program scale-up and spread in low and middle income countries, we describe CHW feedback on: program design and management; community fit and engagement; and integration of the program within the broader health system. Using this framework, we also elicited CHW impressions of program quality and assessed their knowledge of PHC basics and their attitudes regarding the rPHC program.

### Sampling and recruitment

Qualitative and quantitative data were collected during the same study period in the same thirteen health clinics in KSD. Clinics were purposively selected for inclusion in the study, and the 93 CHWs assigned to these clinics were invited to participate in both focus group discussions (FGDs) and a survey on knowledge and attitudes related to rPHC. 91 agreed to participate in the FGDs and survey; the two CHWs who declined to participate did not specify their reasons.

### Data collection

Focus group discussions: FGD guides were translated, back translated, and pre-tested in non-project districts by trained bilingual research assistants. The study team explained the study, invited CHWs to participate, obtained informed consent, and conducted 12 FGDs with a total of 91 CHWs, exploring their outreach activities, perceptions, barriers, and suggestions for program improvement. FGDs were conducted in Xhosa between July and September 2014 and took place in the health facility; each took approximately two hours. Only research staff was present during the time of the discussion. FGDs were audio recorded, translated into English and then transcribed. A bilingual senior research staff member validated each focus group transcript for completeness and accuracy prior to coding.

Knowledge/attitudes surveys: The 91 CHWs who participated in FGDs also completed knowledge and attitudes survey about their job. The self-administered surveys included 131 questions, covering demographics, training, rPHC outreach team experience, job satisfaction, and basic PHC knowledge. The knowledge section included 96 questions from the pre/post-test used by FPD to assess rPHC training in Eastern Cape. All of the completed surveys were entered into a secure Epi Info database, with internal data quality checks for valid entries, skip patterns, range checks, and missing values.

### Data analysis

Qualitative data were entered, cleaned, and analyzed using the NVivo Software Package (Version 10). Audio recordings were translated into English, transcribed by bilingual research assistants, and reviewed for completeness by the bilingual study coordinator. Data were then coded by question and theme. FGD transcripts were analysed in their entirety but a directed approach to content analysis was applied to each transcript [10]. Team members were consulted to reach consensus on data interpretation.

Quantitative survey data were entered, cleaned, and analyzed using Epi Info. First level analysis identified measures of central tendency: means, modes, medians. Second level analysis sought to identify relationships through tabulation of outcome/output variables against basic background characteristics captured at the beginning of each instrument. Aggregate scores from the knowledge component of the KAP survey were compared with aggregate scores from all 330 CHWs taking the FPD test in Eastern Cape in 2012 and 2013.

### Ethical statement

The Eastern Cape Department of Health, the Columbia University Medical Center Institutional Review Board (IRB-AAAN2057), and the Ethics Committee of the University of the Free State (ECUFS NR 22/2014) approved the study protocol. All participants provided written consent prior to their participation in this study and were aware of their right to withdraw at any time. Following explanation of study procedures, risks and benefits, each participant was asked to sign a copy of the consent form; all participants were given copies of the General Information Sheet and Consent Form for their records in addition to contact information of study staff. Both IRBs approved this consent procedure. Participants were not financially incentivized to participate although they were provided with light refreshments during the interview and discussion.

## Results

### Participants

Of the 91 participating CHWs, 90% were female, with a mean age of 41.5 years (range 22–57). Approximately one-third (31%) reported completion of secondary school and 10% reported completion of university education. All but one were born in Eastern Cape Province, 82% had worked as CHWs in KSD sub-district for more than five years, and 67% indicated they had been recruited to be a CHW by a local chief or leader.

### Training, supervision

#### Survey results

Of the 91 CHW who completed the survey, 69% reported rPHC training, 43% reported field-based (“non-classroom”) mentorship, and 13% reported receiving refresher training since they were hired. Participants’ average score on the test of rPHC knowledge was 64.8% (SD 11.9), significantly lower than the pre-test and post-test scores of the 330 CHWs trained in 2012–2013 (p < 0.001).

Although the initial expectation was that supervisors would meet with CHWs weekly and perform field visits at least once a month, when asked about supervision, 48% of CHWs noted that they met with their supervisor once a month or less. Most of these visits were clinic-based; 35% of CHWs reported ever receiving field-based supervision from their nurse team leader. One-fifth (20%) of CHWs reported receiving coaching or skills development from their supervisors, and 8% had received a formal evaluation of their work in the previous 12 months.

Almost half of respondents (45%) strongly agreed and 18% somewhat agreed with the statement: “In general, I am satisfied with this job.” CHWs reported feeling overworked, with less than half (42%) agreeing that their workload was manageable, but 74% agreed that: “if it were up to me, I would continue to work for this organization for quite some time.”

### Scope of work and satisfaction

#### Focus group discussions

In FGDs, participants reported satisfaction with their initial classroom training, and expressed a desire for additional hands-on training and field-based supervision. The majority of FGD participants described what they felt was insufficient supervision, although several couched this with the understanding that team leaders were under-resourced and over-stretched.

As one CHW said,

…the supervision is not sufficient. We would like to go to the field with her [the nurse supervisor]. Even one day would be fine. It’s always us who have to come to the clinic.

Another CHW noted:

We need a team leader who will do home visits with us. The supervisor that we have has never done any home visits with us. We only see her at the end of the month to check on our books. We have incidents that we tell her that need her attention but she tells us that she is busy…and she sometimes tells us that she does not have transport to come. We do not know whether we are doing things correctly because there is no one to guide us.

**Fig 2:**
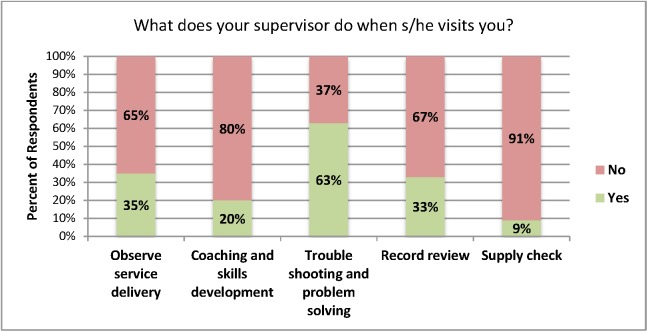
CHW description of supervisory meetings.

CHWs described supervisory meetings as largely focused on issues that required problem-solving skills such as clients’ health problems, transportation challenges and logistical concerns, with less time spent on record review, observation of service delivery and coaching or skills development (Fig 2).

While program design was not an explicit topic of discussion in most FGDs, there was notable tension with regards to CHW goals and objectives, despite the existence of a formal SOW. There was broad agreement amongst respondents that CHWs were intended to act as health educators, providing community members with information about healthy behaviors. Many, but not all CHWs also appreciated that they were intended to facilitate referrals and linkages to clinics. Others felt that they were intended to facilitate health service delivery and to provide “doorstep” services, including medications for HIV and other chronic diseases, although these activities were not part of their official SOW. As two CHWs noted,

CHW should be given more training and tools to be able to do their work well when they are visiting communities and treat people in their homes, [such as] skills and equipment for emergency treatment in the field. We wish we could be able to treat people even before they even go to the clinic but we do not have proper training in the field.

Let me put it this way, you spot a thing, you identify a problem but you have no way to solve it. So the effectiveness of it…I don’t see it.

CHWs felt that community members also had misconceptions about CHW roles and responsibilities, which caused tensions with some community members. As some CHWs mentioned,

What makes CHWs to appear unwelcomed is the fact that when you visit these homes you teach them about health issues. In return they expect you to have a solution on every problem they bring.

We don’t have medication for our clients. They expect us to bring them medication. It is hard when you reach a household you talk and talk but don’t provide anything.

In FGDs, effective health promotion was the most frequently cited success. For example, one CHW said,

What is important is that people like what we are doing and if there is something they do not understand they ask us–the health education is really working.

An example of successful health promotion was the ability of CHWs to support medication adherence and retention in care for community members with HIV, TB and other chronic diseases. As one CHW mentioned,

Since we have started working in the communities, the rate of (medication) defaulters has decreased. We encourage them to take their treatment even though they complain about side effects.

### Community fit

#### Survey results

CHWs felt accepted and appreciated by communities, but were less confident that their services met community needs and expectations. When asked about this topic on the written survey, 13% characterized the fit as excellent, 35% felt it was good, 32% felt it was fair, and 20% felt it was poor.

#### Focus group discussions

In FGDs, CHWs reported engagement and fit with their communities as an important enabling factor. The rPHC engagement process used to foster links with traditional healers, chiefs, and political leaders was perceived as a success, as was the process for CHW selection and the sensitization of community members to the rPHC program. CHWs also described a productive rapport between community members and outreach teams:

Community members see [rPHC] as a good program. Some call to inform us that they cannot come to the clinic for their set dates because of certain problems. So we give them alternative solutions. Some have emergency issues and ask if we could help them to get immediate attendance at the clinic. So they see our importance now, as they talk freely with us, adhere to our programs and follow up on treatment.

Community members are aware of us. When they are not taking their medications and do not want to go to the clinic they come to our houses for us to give them advice or we go to theirs. Then we accompany them to the clinic.

In almost a third of focus groups, CHWs explicitly indicated the importance of building bridges between traditional healers and the formal health system, including engagement and sensitization of traditional healers and chiefs. For example, one CHW discusses the assistance offered to them by community leaders,

Community members appreciate what we are doing, to the extent that if a particular client in a household is running away from us, they even suggest that we must take up the matter to the Chief. The Chief would use his authority and make means to ensure that the case is sorted out.

CHWs reported that they also educated traditional healers about HIV and other illnesses as a means to pass information on to the community members. As one CHW mentioned,

There is a greater level of acceptance of illnesses that used to be associated with stigmatization. This happens to the extent that we even visit traditional healers and educate them about such illnesses.

CHWs who were concerned about meeting community expectations reiterated the challenges regarding their role and scope of work, which they and their clients felt should extend beyond health promotion. CHWs recognized the role they could be playing in their communities by providing basic primary care service delivery like blood pressure checks and medication delivery. As one CHW pointed out,

Elderly people complain that they want their treatment to be brought straight into their homes, but we keep asking them to go the clinics that have no care. So, it would be better if government could equip us with wheelchairs, blood pressure machines, etc. Since we have none of these tools we do not deliver according to our promises, as a result communities lose hope in us.

### Integration within the broader health system

#### Focus group discussions

CHWs perceived advantages of being part of the formal health system, and many saw themselves as agents of change in the communities they serve. When asked, “What was the most satisfying aspect of your work with the outreach team?” one CHW responded,

I think it’s to see the work of the Department of Health moving forward. The clinics are far but when we visit homes and educate about health, people listen and do what we have taught them. When you have taught them about health issues they get encouraged and go to the clinic. Then I get very satisfied as I can see my role as a CHW with the Department of Health moving that agenda forward.

CHWs noted that in some cases they were “competing” with CHWs working for more vertical disease-specific projects run by NGOs. The generalist CHWs on the rPHC outreach teams felt that community members had come to expect more than they could provide. As one CHW highlighted,

As CHWs under government, there are also CHWs from NGOs. So the ones from NGOs bring food parcels (spinach and milk) and now when we as CHWs from the clinics visit community members, they do not even want to listen to what we have to say as we have brought nothing.

CHWs also felt the lack of formal signifiers of their status as DoH staff within the community. Nametags and uniforms were cited as ways to recognize that CHWs were part of the formal health system, and CHWs perceived the lack of such signifiers as undermining their ability to deliver services with authority and credibility.

### Program quality

#### Survey results

**Fig 3:**
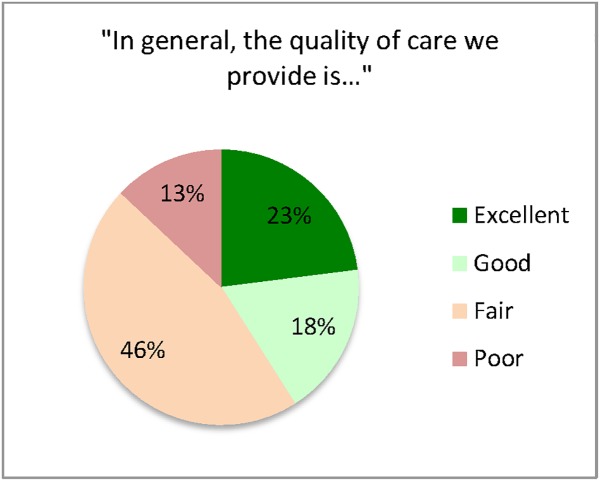
CHW perspectives on quality of rPHC outreach services.

Most (81%) of the CHWs who completed the survey believed that the rPHC outreach teams were effective. More than half raised concerns about the quality of the services they provided. When asked to characterize the quality of community based care provided by their own teams, 46% responded “fair” while 13% responded “poor” (Fig 3). The majority of CHWs (89%) said there were no effective transportation systems to bring clients to health facilities. When asked if they felt they had all of the equipment needed to complete their tasks, 99% responded “no”.

#### Focus group discussions

The majority of focus group participants reported concerns about program quality, with common themes including staffing challenges, insufficient supervision, and lack of transportation, equipment, and supplies (such as medication). As one CHW stated,

…we do not have treatment for people who are on chronic treatment but have medication like panado [acetaminophen] for people who are sick at that moment. We are not given proper medication for us to give people.

Another CHW described staffing challenges at the clinics after they refer patients:

When we do refer people to clinic there is a long queue of people outside and all these people are waiting to be seen by the nurse and there is only one nurse working here. There is a shortage of staff. You find that people leaving the clinic very late which at night only to walk home because of bad transport so it is not safe for them to do so.

Supervision was cited as a major program quality challenge. As one CHW points out:

The supervision is not sufficient. We would like to go to the field with her so she can help us there. Even just one day would be fine. The problem is that she doesn’t have a government vehicle. She uses her own car. It’s always us who have to come to the clinic.

Many CHWs described negative community attitudes directed at CHWs due to a lack of needed medical supplies, as one CHW says:

People have developed negative attitudes towards us. They are fed up on us because we do not have the needed resources.

The lack of transportation posed two barriers for CHWs, limiting the ability of outreach teams to access communities, while also making it difficult for them to effectively refer patients to health facilities. Once CHWs reached communities, distance, bad roads, and lack of transportation limited their ability to facilitate referrals and linkages when they identified patients in need of care. As one CHW described it,

There is no proper transport but people are determined to visit the clinic, we are even struggling to go there and they do not bring their children for immunization.

Given the difficulties getting patients to health facilities, CHWs on outreach teams were eager to bring treatment to communities, in addition to health education and advice. While some were empowered to deliver medications to patients with chronic illnesses, CHWs reported that this was rare, due to inadequate systems, planning, and coordination with clinic staff. The lack of equipment, supplies and transportation put CHWs in a difficult position, unable to address health needs or to provide effective referrals. In FGDs, insufficient practical training and resource materials and tools were linked with the perception of poor outcomes.

## Discussion

Community health workers are often deployed in low-resource settings due to heath worker shortages and as a means of providing effective and contextually appropriate services in both urban and rural settings [18–19]. The need to carefully optimize CHW program design and implementation has been evident for decades, however. The expansion of national CHW programs following the 1978 Alma Ata declaration was followed by substantive problems due to poor planning and resourcing, unrealistic expectations, overwhelming workloads and poor management [11–12]. Critical reviews of large-scale CHW programme successes and failures highlight many of the issues discussed by CHWs in this study [11, 13–17].

More recently, in response to an often-fragmented CHW landscape resulting from NGO-implemented, disease-specific CHW programs, there has been growing interest in standardizing CHW portfolios and integrating them into national health systems, as South Africa has done in the context of its rPHC initiative. While barriers and facilitators to the scale-up and integration of CHWs have been studied in multiple contexts, the perspective of CHWs themselves has not been frequently explored [20–24].

The CHWs we studied enjoyed their work and found it meaningful. They also suggested ways to strengthen the implementation of outreach team oversight, strongly recommending field-based training and more supervision in the community, rather than retrospective clinic-based reviews by nurses. CHW test scores indicated a likely fall-off in knowledge over time, and suggest the need for on-going refresher trainings and mentorship. These findings are consistent with studies showing that effective CHW supervision can be an important motivator [25], and that CHW supervisors are infrequently trained in mentoring or coaching, often have conflicting responsibilities, and rarely have the capacity to travel to the communities where the CHWs work [26]. Staff shortages and inadequate resources for supervisory activities and competing demands on supervisors’ time may also contribute to implementation failure for CHW programs [27].

CHWs also struggled with their roles and scope of work, perceiving tensions between community needs and expectations and the services they were equipped and authorized to deliver. Challenges related to differing expectations, and unclear or inappropriate scopes of work for CHWs have also been noted in the literature [28–30].The rPHC outreach teams in KSD were tasked with providing a bridge between facilities and communities, but CHWs noted their inability either to bring medications and health services to the community, or to improve access to health facilities for clients in remote locations. Many of the CHWs in this study indicated that the ability to deliver medication refills, and the authority to monitor measures such as blood pressure, weight, and patient wellbeing would enhance the impact and acceptance of rPHC outreach teams, as well as the morale of the CHWs themselves. Pallas et al. suggest that the design of effective CHW programs requires a nuanced understanding of which health services the community perceives as most valuable [9]. While health promotion activities are rarely rejected, curative services may be perceived as more important to community members, and interventions such as treatment of pneumonia and malaria, provision of vitamin A, and nutrition support may improve CHW acceptance and credibility [9].

The advantages and disadvantages of CHW integration into the formal health system were clear to study participants, who identified their broad mandate and links to DOH clinics as a strength, and the fact that they were less well-resourced than “competing” disease-focused CHW programs as a challenge. Their interest in signifiers of their official status–name tags and/or uniforms–echoes the literature that suggests CHWs derive important motivation from various non-financial incentives such as uniforms, nametags, and certificates [22, 30].

CHWs were thoughtful in terms of their assessment of the quality of the services they provide. CHWs identified insufficient field based practical training, suboptimal supervision, and lack of equipment and supplies, and transportation barriers as important problems. Their scores on the PHC test also indicated a possible attenuation of knowledge over time, and the need for ongoing refresher trainings and mentoring. Evidence on the quality of CHW services in low-resource settings is mixed [24]. In a recent Cochrane review, CHWs often attributed poor quality services to insufficient training [30]. Evaluations of disease specific programs have noted that appropriate training, clear understandings of roles and responsibilities, and regular supportive supervision are important facilitators of successful CHW health service delivery [31].

This study emphasizes the importance of soliciting the perspective and voices of those responsible for the implementation of a new primary health care program. This analysis highlights the perception of CHWs launching a new primary health care initiative, and contributes to the literature on CHW assessment of barriers and facilitators to program success.

Our analysis had several limitations. The use of convenience sampling and focus on one sub-district limits the generalizability of the findings, although they do align with previously published studies. Data collection occurred in health facilities, and while no clinic staff or supervisors were present, this may have constrained participants from reflecting critically on the health services they were being paid to provide. In addition, while we report CHW perceptions of program quality, the study did not measure actual program quality and additional research may be needed to determine optimal approaches to program design and delivery. The perspectives of other key WBOT stakeholders, such as nurse supervisors, community leaders and program implementers, should be a subject of further research.

## Conclusions

In conclusion, this study explored the knowledge and perceptions of CHWs in the first year of the rPHC initiative in the Eastern Cape Province. Overall, CHWs felt that rPHC has the potential to transform community health. They valued the robust, thorough community consultations that preceded project launch, noting the positive impact on buy-in and trust on the part of community members, traditional healers, and community leaders. The CHWs also highlighted issues in program management, supervision, scope and quality that challenged their ability to deliver on the potential of CHWs for improving community health and wellbeing.

## References

[1] Pillay Y, Barron P. The implementation of PHC re-engineering in South Africa. Public Health Association of South Africa; 2011 Available: http://www.phasa.org.za/the-implementation-of-phc-re-engineering-in-south-africa/

[2] National Department of Health. Re-engineering primary health care in South Africa: Discussion document. Pretoria: Department of Health; 2010.

[3] Schneider H, Hlophe H, van Rensburg D, Community health workers and the response to HIV/AIDS in South Africa: tensions and prospects Health Policy Plan. 2008, 23 (3):179–187. 10.1093/heapol/czn006 18388133

[4] Van Ginneken N., Lewin S., & Berridge V. The emergence of community health worker programmes in the late apartheid era in South Africa: An historical analysis. Social Science & Medicine (1982). 2010; 71(6–3), 1110–1118. 20638169 10.1016/j.socscimed.2010.06.009 PMC2941026

[5] Nxumalo N., Goudge J., & Thomas L. Outreach services to improve access to health care in South Africa: lessons from three community health worker programmes. Global Health Action. 2013; 6, 10.3402/gha.v6i0.19283. 10.3402/gha.v6i0.19283 PMC3556683 23364101

[6] Massyn N, Day C, Peer N, Padarath A, Barron P, English R, editors. District Health Barometer 2013/14. Durban: Health Systems Trust; 10 2014 Available: http://www.health-e.org.za/2014/10/29/report-district-health-barometer-2013-14/

[7] King Sabata Dalindyebo Local Municipality: Draft IDP Review 2014/15. Available: http://mfma.treasury.gov.za/Documents/01.%20Integrated%20Development%20Plans/2014-15/02%20Local%20Municipalities/EC157%20King%20Sabata%20Dalindyebo/EC157%20King%20Sabata%20Dalindyebo%20Draft%20IDP%20Budget%20Review%202014-15.pdf [accessed 25 April 2015]

[8] Rabkin M, Mutiti A, Mwansa J, Macheka T, Austin-Evelyn K, El-Sadr WM. Re-engineering primary health care: a formative process evaluation of rPHC implementation in King Sabata Dalundyebo sub-district in the Eastern Cape Province of South Africa. 2015. Available: http://academiccommons.columbia.edu/catalog/ac:192781

[9] Pallas SW, Minhas D, Perez-Escamilla R, Taylor L, Curry L, Bradley EH. Community health workers in low- and middle-income countries: what do we know about scaling up and sustainability? American journal of public health. 2013;103(7):e74–82. 10.2105/AJPH.2012.301102 23678926 PMC3682607

[10] Hsieh H. F., & Shannon S. E. Three approaches to qualitative content analysis. Qualitative health research, 2005, 15, 1277–1288. 10.1177/1049732305276687 16204405

[11] Lehmann US, D (2007) Community health workers: What do we know about them? The state of the evidence on programmes, activities, costs and impact on health outcomes of using community health workers. University of the Western Cape.

[12] Singh PS, Jeffrey D (2013) 1 million community health workers in sub-Saharan Africa by 2015. diabetes 14: 17. 10.1016/S0140-6736(12)62002-9 23541538

[13] Gilson LW G.; Heggenhougen K.; Owuor-Omondi L.; Perera M.; Ross D.; Salazar L. (1989) National community health worker programs: how can they be strengthened? J Public Health Policy 10: 518–532. 2621254

[14] Tulenko KM, S.; Afzal M. M.; Frymus D.; Oshin A.; Pate M.; Quain E.; Pinel A.; Wynd S.; Zodpey S. (2013) Community health workers for universal health-care coverage: from fragmentation to synergy. Bull World Health Organ 91: 847–852. 10.2471/BLT.13.118745 24347709 PMC3853952

[15] Samb BC F.; Holloway J.; Van Damme W.; De Cock K. M.; Dybul M. (2007) Rapid expansion of the health workforce in response to the HIV epidemic. N Engl J Med 357: 2510–2514. 10.1056/NEJMsb071889 18077816

[16] Tendler J (1997) Preventative Health: The case for the unskilled meritocracy Good government in the Tropics. Baltimore: Johns Hopkins University Press pp. 21–45.

[17] Lewin S, Munabi-Babigumira S, Glenton C, Daniels K, Bosch-Capblanch X, van Wyk BE, et al Lay health workers in primary and community health care for maternal and child health and the management of infectious diseases. Cochrane System Rev. 2010;3:CD004015. 10.1002/14651858.CD004015.pub3 PMC6485809 20238326

[18] Gilmore B, McAuliffe E. Effectiveness of community health workers delivering preventive interventions for maternal and child health in low-and middle-income countries: a systematic review. BMC Public Health. 2013;13:847 10.1186/1471-2458-13-847 24034792 PMC3848754

[19] Hill Z, Dumbaugh M, Benton L, Kallander K, Strachan D, Ten Asbroek A, et al Supervising community health workers in low-income countries—a review of impact and implementation issues. Global health action. 2014;7:24085 10.3402/gha.v7.24085 24815075 PMC4016747

[20] Strachan DL, Kallander K, Ten Asbroek AH, Kirkwood B, Meek SR, Benton L, et al Interventions to improve motivation and retention of community health workers delivering integrated community case management (iCCM): stakeholder perceptions and priorities. The American journal of tropical medicine and hygiene. 2012;87(5 Suppl):111–9. 10.4269/ajtmh.2012.12-0030 23136286 PMC3748511

[21] Bhattacharyya K, Winch P, LeBan K, Tien M. Community Health Worker Incentives and Disincentives: How They Affect Motivation, Retention, and Sustainability. Arlington, Virginia: Basic Support for Institutionalizing Child Survival Project (BASICS II) for the United States Agency for International Development; 2001.

[22] Kok MC, Dieleman M, Taegtmeyer M, Broerse JE, Kane SS, Ormel H, et al Which intervention design factors influence performance of community health workers in low- and middle-income countries? A systematic review. Health Policy Plan. 2014. 10.1093/heapol/czu126 PMC4597042 25500559

[23] Glenton C, Scheel IB, Pradhan S, Lewin S, Hodgins S, Shrestha V. The female community health volunteer programme in Nepal: decision makers' perceptions of volunteerism, payment and other incentives. Social science & medicine. 2010;70(12):1920–7. Epub 20382464 10.1016/j.socscimed.2010.02.034

[24] Akintola O, Chikoko G. Factors influencing motivation and job satisfaction among supervisors of community health workers in marginalized communities in South Africa. Human Resources for Health 2016;14:54 10.1186/s12960-016-0151-6 27601052 PMC5013625

[25] Berman P, Franco L. Formal Health System Support Activities and Community Health Worker Performance. Global Health Evidence Summit: Community and Formal Health System Support for Enhanced Community Health Worker Performance. Washington, DC; 2012.

[26] Ndima SD, Sidat M, Give C, Ormel H, Kok MC, Taegtmeyer M. Supervision of community health workers in Mozambique: a qualitative study of factors influencing motivation and programme implementation. Human Resources for Health. 2015; 13:63 10.1186/s12960-015-0063-x 26323970 PMC4556309

[27] Raven J, Akweongo P, Baba A, Olikira Baine S, Gulaye Sall M, Buzuzi S, Martineau T. Using a human resource management approach to support community health workers: experiences from five African countries. Human Resources for Health. 2015 13: 45 10.1186/s12960-015-0034-2 26324423 PMC4556018

[28] Lehmann U, Sanders D. Community health workers: What do we know about them? The state of the evidence on programmes, activities, costs and impact on health outcomes of using community health workers Geneva: Evidence and Information for Policy, Department of Human Resources for Health, World Health Organisation, 2007

[29] Haile F, Yemane D, Gebreslassie A. Assessment of non-financial incentives for volunteer community health workers–the case of Wukro district, Tigray, Ethiopia. Human Resources for Health. 2014;12:54 10.1186/1478-4491-12-54 25245633 PMC4180263

[30] Harvey SR, Fernandez ME. Identifying the Core Elements of Effective Community Health Worker Programs: A Research Agenda. American Journal of Public Health. 2012;102(9):1633–1637. 10.2105/AJPH.2012.300649 22813092 PMC3415604

[31] Smith Paintain L, Willey B, Kedenge S, Sharkey A, Kim J, Buj V, Webster J, Schellenberg D, Ngongo N. Community health workers and stand-alone or integrated case management of malaria: a systematic literature review. American Journal of Tropical Medicine. 2014: 91(3):461–70 10.4269/ajtmh.14-0094 PMC4155545 24957538

